# Supplementary material for: Progression of coronary artery calcification in conventional hemodialysis, nocturnal hemodialysis, and kidney transplantation
Source: PLoS One. 2020 Dec 30;15(12):e0244639. doi: 10.1371/journal.pone.0244639 (PMC7773242; doi:10.1371/journal.pone.0244639)
Supplement: S1 Table — (DOCX) [file pone.0244639.s001.docx]

**S1 Table.** **Characteristics at enrollment of 114 patients who completed at least one follow-up visit and of 36 who did not.**

|  | **Study population**  **(n = 114)** | **Did not complete follow-up visit (n = 36)** |
| --- | --- | --- |
| ***Demographics and medical history*** |  |  |
| **Age (years)** | 53 ±13 | 52 ±13 |
| **Male sex (%)** | 75 (66%) | 23 (64%) |
| **Diabetes mellitus (%)** | 14 (12%) | 10 (28%) |
| **Cardiovascular disease (%)** | 22 (19%) | 10 (28%) |
| **Current smoker (%)** | 16 (14%) | 6 (17%) |
| ***History of kidney disease*** |  |  |
| **Dialysis duration (months)** | 28 (12–56) | 36 (18–66) |
| **Cause of end-stage renal disease (%)** |  |  |
| - **Cystic kidney disease** | 24 (21%) | 1 (3%) |
| - **Interstitial nephritis** | 3 (3%) | 3 (8%) |
| - **Glomerulonephritis** | 28 (25%) | 13 (36%) |
| - **Vascular disease** | 22 (19%) | 9 (25%) |
| - **Diabetic nephropathy** | 9 (8%) | 3 (8%) |
| - **Other** | 14 (12%) | 4 (11%) |
| - **Unknown** | 14 (12%) | 3 (8%) |
| ***Renal replacement therapy*** |  |  |
| **Conventional hemodialysis (%)** | 32 (28%) | 25 (69%) |
| **Nocturnal hemodialysis (%)** | 34 (30%) | 5 (14%) |
| **Kidney transplantation (%)** | 48 (42%) | 6 (17%) |
| ***Medication use*** |  |  |
| **Vitamin K antagonists (%)** | 16 (14%) | 4 (11%) |
| **Vitamin D analogs (%)** | 51 (45%) | 24 (67%) |
| **Calcium-containing phosphate binder (%)** | 48 (42%) | 10 (28%) |
| **Cinacalcet (%)** | 20 (18%) | 11 (31%) |
| ***Physical and laboratory parameters*** |  |  |
| **Body mass index (kg/m^2^)** | 25.5 ±4.6 | 26.7 ±4.8 |
| **Systolic blood pressure (mmHg)** | 135 ±18 | 137 ±19 |
| **Diastolic blood pressure (mmHg)** | 78 ±10 | 77 ±11 |
| **Calcium (mmol/L)** | 2.3 ±0.1 | 2.3 ±0.2 |
| **Albumin (g/L)** | 40.7 ±3.4 | 40.4 ±3.0 |
| **Phosphate (mmol/L)** | 1.2 ±0.5 | 1.6 ±0.5 |
| **Parathyroid hormone (pmol/L)** | 16 (6–35) | 22 (11–45) |

Data are presented as mean ±standard deviation, median (interquartile range) or number (percentage).

Data of patients on nocturnal hemodialysis and kidney transplant recipients were measured at enrollment, i.e. about 3 months after initiating this treatment.

Abbreviations: eGFR: estimated glomerular filtration rate, calculated with the Chronic Kidney Disease-Epidemiology Collaboration equation 2009.
