# Supplementary material for: Progression of coronary artery calcification in conventional hemodialysis, nocturnal hemodialysis, and kidney transplantation
Source: PLoS One. 2020 Dec 30;15(12):e0244639. doi: 10.1371/journal.pone.0244639 (PMC7773242; doi:10.1371/journal.pone.0244639)
Supplement: S2 Table — (DOCX) [file pone.0244639.s002.docx]

**S2 Table. Agatston scores at annual follow-up exams in 114 patients with end-stage renal disease.**

|  | ***N**** | **Inclusion** | ***N*** | **Year 1** | ***N*** | **Year 2** | ***N*** | **Year 3** |
| --- | --- | --- | --- | --- | --- | --- | --- | --- |
| **Conventional hemodialysis** | *32* | 206 (1–897) | *32* | 234 (20–1041) | *21* | 103 (7–890) | *11* | 426 (81–852) |
| **Nocturnal hemodialysis** | *34* | 165 (18–586) | *34* | 269 (12–751) | *24* | 494 (51–780) | *19* | 602 (131–880) |
| **Kidney transplantation** | *48* | 112 (6–606) | *48* | 138 (20–612) | *44* | 131 (28–727) | *42* | 178 (39–824) |

Agatston scores are presented as median (IQR).

*Patients without any follow-up exams were not included in the current analyses.
