# Supplementary material for: Progression of coronary artery calcification in conventional hemodialysis, nocturnal hemodialysis, and kidney transplantation
Source: PLoS One. 2020 Dec 30;15(12):e0244639. doi: 10.1371/journal.pone.0244639 (PMC7773242; doi:10.1371/journal.pone.0244639)
Supplement: S3 Table — (DOCX) [file pone.0244639.s003.docx]

**S3 Table. Longitudinal changes in Agatston scores between annual follow-up exams in 114 patients with end-stage renal disease.**

|  | **N** | **Mean change**  **per year** | **Unadjusted difference** | **Model 1*** | **Model 2^†^** | **Model 3^‡^** |
| --- | --- | --- | --- | --- | --- | --- |
| **Conventional hemodialysis** | 32 |  |  |  |  |  |
| ΔCAC SQRA |  | 1.48  (0.86 to 2.10) | 0.0  *(reference)* | 0.0  *(reference)* | 0.0  *(reference)* | 0.0  *(reference)* |
| **Nocturnal hemodialysis** | 34 |  |  |  |  |  |
| ΔCAC SQRA |  | 1.31  (0.73 to 1.88) | -0.17  (-1.02 to 0.68) | -0.02  (-0.77 to 0.73) | -0.03  (-0.77 to 0.70) | 0.06  (-0.70 to 0.84) |
| **Kidney transplantation**** | 48 |  |  |  |  |  |
| ΔCAC SQRA |  | 0.93 (0.48 to 1.39) | -0.55  (-1.32 to 0.22) | -0.32  (-1.01 to 0.36) | -0.30  (-0.97 to 0.37) | 0.07  (-0.75 to 0.91) |

95% confidence intervals between brackets.

*Model 1 = Adjusted for square root transformed Agatston score (CAC SQRA) at inclusion.

^†^Model 2 = Model 1 + age and sex.

^‡^Model 3 = Model 2 + diabetes mellitus, dialysis duration, current smoking, presence of residual urine production, and vitamin K antagonist use.

**adjusted difference in ΔCAC SQRA between kidney transplant recipients and patients on peritoneal dialysis: -1.42, 95% CI -2.36 to -0.46)
