# Supplementary material for: Progression of coronary artery calcification in conventional hemodialysis, nocturnal hemodialysis, and kidney transplantation
Source: PLoS One. 2020 Dec 30;15(12):e0244639. doi: 10.1371/journal.pone.0244639 (PMC7773242; doi:10.1371/journal.pone.0244639)
Supplement: S1 Fig — (DOCX) [file pone.0244639.s005.docx]

**
S1 Fig.** Phosphate levels during follow-up in 114 patients with end-stage renal disease, stratified by renal replacement therapy.
