# Supplementary material for: Progression of coronary artery calcification in conventional hemodialysis, nocturnal hemodialysis, and kidney transplantation
Source: PLoS One. 2020 Dec 30;15(12):e0244639. doi: 10.1371/journal.pone.0244639 (PMC7773242; doi:10.1371/journal.pone.0244639)
Supplement: S1 File — (PDF) [file pone.0244639.s006.pdf]

PROTOCOL TITLE

Is progression of arteriosclerosis in ESRD patients inhibited by nocturnal hemodialysis or renal transplantation?

versie 6, 05-02-2018

Protocol ID	NOCTX
Short title	Arterial calcifications in nocturnal hemodialysis and renal transplantation vs. conventional dialysis
Version	6
Date	05 February 2018
Coordinating investigator/project leader	Dr. B.C. van Jaarsveld<br>Dianet Dialysecentra, locatie Utrecht<br>Brennerbaan 130<br>3524 BN Utrecht<br>tel + 31 30 880 8300
Principal investigators	UMC Utrecht<br>Heidelberglaan 100<br>3508 GA Utrecht<br>tel + 31 88 755 8604<br>Drs. T.T. Jansz
Sponsors (Dutch: verrichter/opdrachtgever)	Dianet Dialysecentra, locatie Utrecht<br>Brennerbaan 130<br>3524 BN Utrecht<br>tel + 31 30 880 8300<br>*Medical director: dr. F.T.J. Boereboom*
Independent physician(s)	Dr. A.D. van Zuilen, internist-nephrologist<br>UMC Utrecht
Laboratory sites	Laboratory for Clinical Chemistry and Hematology – Research, University Medical Center Utrecht

PROTOCOL SIGNATURE SHEET

Name	Signature	Date
Sponsor or legal representative:	Chair of Executive Board<br>Dianet Dialysecentra<br>Mw. drs. J.W. Hingst	05/03/2018
Head of Department:	Medical Director<br>Dianet Dialysecentra<br>Dr. F.T.J. Boereboom	05/03-2018
	Head of Department of<br>Nephrology, UMC Utrecht<br>Mw. Prof. dr. M.C. Verhaar	06/03/2018
Coordinating Investigator/Project Leader	Coordinating<br>Investigator/Project Leader<br>Dianet Dialysecentra<br>Dr. B.C. van Jaarsveld	8-2-18
Principal Investigator	UMC Utrecht<br>Drs. T.T. Jansz	08/02/18

TABLE OF CONTENTS

	Page
Summary	
1. Introduction and rationale	6
2. Objectives	8
3. Study design	10
4. Study population	11
5. Treatment of subjects	12
6. Investigational medicinal product	14
7. Methods	16
8. Safety procedures	17
9. Statistical analysis	21
10. Ethical considerations	22
11. Administrative aspects and publication	23
12. References	25
	26

LIST OF ABBREVIATIONS AND RELEVANT DEFINITIONS

ABR	ABR form (General Assessment and Registration form) i.e. the application form required for submission to the accredited Ethics Committee (ABR = Algemene Beoordeling en Registratie)
AE	Adverse Event
AR	Adverse Reaction
CA	Competent Authority
CCMO	Central Committee on Research Involving Human Subjects
CV	Curriculum Vitae
DSMB	Data Safety Monitoring Board
EU	European Union
EudraCT	European drug regulatory affairs Clinical Trials
GCP	Good Clinical Practice
IB	Investigator's Brochure
IC	Informed Consent
IMP	Investigational Medicinal Product
IMPD	Investigational Medicinal Product Dossier
METC	Medical research ethics committee (MREC); in Dutch: medisch ethische toetsings-commissie (METC)
(S)AE	(Serious) Adverse Event
SPC	Summary of Product Characteristics (in Dutch: officiële productinformatie IB1-tekst)
Sponsor	The sponsor is the party that commissions the organization or performance of the research, for example a pharmaceutical company, academic hospital, scientific organization or investigator. A party that provides funding for a study but does not commission it is not regarded as the sponsor, but referred to as a subsidizing party.
SUSAR	Suspected Unexpected Serious Adverse Reaction
WBP	Personal Data Protection Act (in Dutch: Wet Bescherming Persoonsgegevens)
WMO	Medical Research Involving Human Subjects Act (Wet Medisch-wetenschappelijk Onderzoek met Mensen)

SUMMARY

Rationale:

Cardiovascular disease is the leading cause of mortality in patients with end-stage renal disease (ESRD). The vascular abnormalities in ESRD are especially characterized by arterial wall calcifications, whereas intimal hyperplasia seen with "classic atherosclerosis" is less pronounced. These calcifications are strongly associated with increased mortality. The exact mechanism by which calcification develops is unknown, but there is a direct relationship with increased serum calcium and serum phosphorus. With long and frequent hemodialysis, such as nocturnal hemodialysis, and with renal transplantation, calcium and phosphate levels can be normalized. It is unknown whether these treatments inhibit the progression of arterial calcification or bring about regression of calcification. The hypothesis of this study is that with nocturnal hemodialysis the progression of arterial calcification is less than with conventional hemo- or peritoneal dialysis, and approximates the progression rate seen with a renal transplant.

Objective:

1. To assess whether nocturnal hemodialysis and renal transplantation are associated with less progression of coronary arterial calcification, compared with conventional hemodialysis and peritoneal dialysis;
2. To identify risk factors for coronary arterial calcification in dialysis and transplant patients, and to assess a possible interaction with treatment modality.

Study design:

Prospective observational cohort study, comparing 4 cohorts with a follow-up of 3 years; each cohort exists of 40-55 patients to account for a 25% lost-to-follow-up-rate (e.g. by renal transplantation in the 3 dialysis groups, or by mortality).

Study population:

A. Patients with ESRD, 18-75 yr, who start treatment with nocturnal hemodialysis (5-7 times/week, 7-8 hr/night);
B. Patients with ESRD, 18-75 yr, who undergo a living or cadaveric renal transplant;
C. Patients with ESRD, 18-75 yr, treated with conventional hemodialysis (3-4 times/week, 3-5 hr);
D. Patients with ESRD, 18-75 yr, treated with conventional peritoneal dialysis.

"Intervention" (different modalities of renal replacement therapy, chosen by the patient, are observed):

Group A: treatment with frequent nocturnal hemodialysis;

Group B: treatment with renal transplantation;

Group C: continuous treatment with hemodialysis;

Group D: continuous treatment with peritoneal dialysis.

Main study parameters/endpoints:

1. Change in coronary artery calcification score, per patient;
2. Change in pulse wave velocity, per patient.

Nature and extent of the burden and risks associated with participation, benefit and group relatedness:

- The study is an observational study, not an intervention-study.
- Participation does not influence or change the nature of the treatment, i.e. the modality of renal replacement therapy;
- Participation is associated with limited physical examination, questionnaires and blood sampling;
- Blood sampling is performed in combination with regular blood sampling, and requires a maximum of 20 cc blood per visit extra, once a year;
- Multislice CT-scan is performed at inclusion, and after 1, 2 and 3 years; this scan takes < 1 minute of time and radiation exposure is less than with conventional abdominal CT-scan; at inclusion and after 3 years, multislice CT-scan with intravenous contrast is performed to examine potential coronary stenosis.
- Yearly measurements of pulse-wave velocity are done with ultrasound (non-invasive), which takes 30 minutes of time.

1. INTRODUCTION AND RATIONALE

Cardiovascular disease (CVD) is the leading cause of death in patients with end-stage renal disease (ESRD)[1]. From several perspectives, the pathophysiology of ischemic cardiovascular events is different in ESRD compared to the general population. In patients with ESRD, the classical risk factors for CVD, such as hypertension, obesity and hyperlipidemia are associated with decreased mortality instead of increased mortality ("reverse epidemiology ")[2-5]. Vascular calcification appears to be the central finding in the pathophysiology of CVD, manifesting itself in increased arterial stiffness and mediasclerosis[3,6]. The presence and extent of vascular calcifications are strong predictors of all-cause and cardiovascular mortality in hemodialysis patients, independent of classical atherogenic factors[3,7].

Elevated calcium and phosphate concentrations or loads in the serum of ESRD patients have direct effects on vascular smooth muscle cells that induce an osteogenic "bone-like" differentiation of these cells[8]. Reducing the calcium concentration in ESRD patients by the prescription of non-calcium-containing phosphate binders can slow down the progression rate of arterial calcification[9,10]. Coronary artery calcification in young hemodialysis patients is strongly associated with dialysis vintage, calcium-phosphorus-product and oral calcium load[11].

It has been well documented that correction of calcium and phosphorus parameters is also possible by means of long and frequent hemodialysis, such as nocturnal hemodialysis[12-14]. A single short-term study on nocturnal hemodialysis showed stabilization of coronary artery calcification score, but comparison with renal transplantation and conventional hemodialysis and peritoneal dialysis was not made[15].

Therefore this study aims to compare the progression of coronary artery calcification score in 4 models of treatment of ESRD: conventional hemodialysis, peritoneal dialysis, renal transplantation and nocturnal hemodialysis.

A second question that is unanswered so far, is the pathophysiology of the vascular calcification in ESRD patients. In ESRD, serum is oversaturated with calcium and phosphorus, and in vitro precipitation would occur with the present calcium and phosphorus concentrations. Extracellular calcium-regulatory proteins, such as matrix gla protein (MGP), osteoprotegerin and human fetuin-A, are potent inhibitors of calcium-phosphorus precipitation[8]. Animals lacking these proteins die at young age due to arterial calcifications and vessel ruptures[16-18]. The serum concentration of fetuin-A is diminished in ESRD[8], and rises after a renal transplant[19]. Fetuin-A-deficiency is an inflammation-related mortality risk factor and the possible missing link between chronic inflammation and high incidence of cardiovascular events in dialysis patients[20]. However, contradictory reports describe the course of fetuin-A deficiency in renal insufficiency and diabetic nephropathy[8,21,22]. Regarding MGP, an inverse relation between undercarboxylated MGP and aortic augmentation index, a marker of vascular stiffness, was found, suggesting that low MGP levels are a marker of active calcification[23].

Therefore this study aims to study non-classical risk factors in patients with ESRD, such as the calcium-regulatory proteins and inflammatory parameters. We expect that patients with advanced age, long period on dialysis, a high calcium phosphate product, increased inflammation markers and deficiency of calcification inhibitors will show the highest progression of coronary calcification. How these factors interact with the treatment modalities under study, remains to be established.

The primary endpoint of this study is the change in coronary calcification score, visualized by multi-slice CT scanning. With this technique, the amount of calcification in coronary arteries can be quantified during 3 heartbeats (i.e. in about 10 seconds) with radiation exposure less than a conventional CT-scan. The burden of this technique is therefore minimal for the patient[24-29].

2. OBJECTIVES

Primary objective:

1. To assess whether frequent nocturnal hemodialysis and renal transplantation are associated with less progression of coronary artery calcifications, in comparison with conventional 3-times/week hemodialysis and with peritoneal dialysis.

Secondary objectives:

1. To assess whether frequent nocturnal hemodialysis and renal transplantation are associated with less progression of coronary artery stenoses, in comparison with conventional 3-times/week hemodialysis and with peritoneal dialysis;
2. To assess the association of calcium-homeostasis, in particular calcium, phosphate, PTH, and relevant medication, with the progression of coronary arterial calcifications and stenoses;
3. To assess the association of markers of inflammation, in particular CRP, interleukin-6, interleukin-1, von Willebrand factor, fibrinogen, with the progression of coronary arterial calcifications and stenoses;
4. To assess the association of calcification-inhibitors, in particular fetuin A, osteoprotegerin and matrix-Gla protein, with the progression of coronary arterial calcifications and stenoses.
5. To assess possible interaction of the abovementioned risk factors and the treatment modalities mentioned in the primary objective.

3. STUDY DESIGN

The study is a prospective cohort study, with a duration of follow-up of 3 years. The design is schematically depicted in the following flow charts.

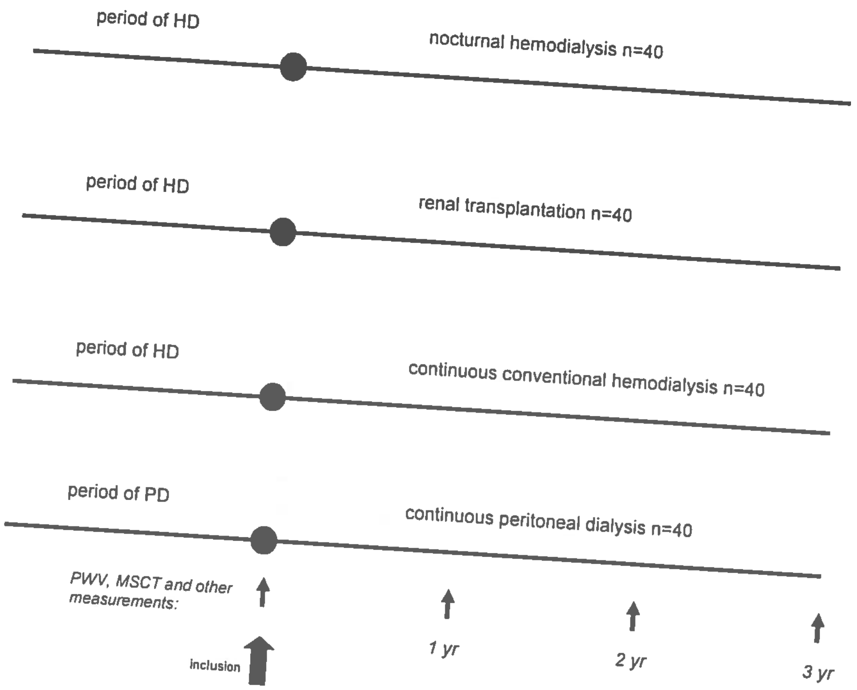

Investigations:

time	*incl*	*½ yr*	*1 yr*	*1 ½ yr*	*2 yr*	*2 ½ yr*	*3 yr*
Multi-slice CT scan (MSCT) of heart	x		x		x		x
Coronary MSCT	x						x
Pulse-wave velocity	x		x		x		x
Lab assessments	x	x	x	x	x	x	x
Weight/Blood pressure	x		x		x		x
Medication	x		x		x		x
Quality-of-life	x		x		x		x
Karnofsky perf. status	x		x		x		x
Clinical events	continuously						

4. STUDY POPULATION

4.1 Population

The study populations consists of 4 cohorts of patients: patients who start with frequent nocturnal hemodialysis, patients who receive a renal transplant and 2 control groups treated with hemodialysis and with peritoneal dialysis. The study is sponsored by Dianet Dialysis Centers, while all research activities take place in University Medical Center Utrecht. Patients were screened at their local treatment facility (Dialysecentrum Groningen, Groningen; Dialysecentrum Diapriva, Amsterdam; VU medisch centrum, Amsterdam; Meander Medisch Centrum, Amersfoort; Rijnstate Ziekenhuis, Arnhem; Sint Antoniusziekenhuis, Nieuwegein; Stichting Dianet Dialysecentra, Utrecht and Amsterdam; and University Medical Center Utrecht, Utrecht), and if interested in the study, subsequently referred to University Medical Center Utrecht for study participation.

- 40 patients who start treatment with frequent nocturnal hemodialysis are asked to participate in the study;
- 40 patients who receive a renal transplant, after having been treated with hemo- or peritoneal dialysis, are asked to participate in the study;
- 40 patients who are treated with conventional hemodialysis are asked to participate; in order to enhance similarity between groups only hemodialysis patients are recruited that are or could be eligible to be on the waiting list for renal transplantation;
- 40 patients who are treated with peritoneal dialysis are asked to participate; in order to enhance similarity between groups only peritoneal dialysis patients are recruited that are of could be eligible to be on the waiting list for renal transplantation.

Groups will be matched according to age, sex, presence of diabetes mellitus and duration of dialysis, in order to minimize between-group differences to 25% regarding these factors.

4.2 Inclusion criteria

- current age > 18 and < 75 yr
- ability to understand the study procedures
- willingness to provide written informed consent

4.3 Exclusion criteria

- life expectancy < 3 months
- claustrophobia
- treatment incompliance, i.e. non-adherence to dialysis regimens and drug use
- GFR < 30 ml/min (according to MDRD formula) in renal transplant patients
- pregnancy

4.4 Sample size calculation

The effects of the different modalities of renal replacement therapy on arterial calcification are analyzed with linear regression, after transformation of calcium scores. The effects of risk

factors for calcification, including inflammation parameters and calcification inhibitors are analyzed in a multivariate model. The mean (±SD) coronary calcification score in ESRD patients is 2370 (± 352)[30]. The multislice CT-scan is extensively validated in our institute. At this level of calcification the variation between independent measurements is less than 10%. Therefore we can demonstrate an increase or decrease in calcification score of 15%, in 4 subpopulations of 22 patients with ESRD, with a type I error probability for a two sided test of 0.05 and a power of 0.90 [formula: "$N = (2(Z\alpha+Z\beta)^2*sd)/(0.15*mean)$ " where N = number of patients per group, $Z\alpha = 1.96$, $Z\beta = 1.28$, S = 3.52, mean = 2370].
To account for a dropout of patients by renal transplantation, switch to a different treatment modality, refusal of further participation or death we intend to include 40-55 patients per group – depending on the dropout, aiming to obtain complete study data in 22 patients per group.

5. TREATMENT OF SUBJECTS

5.1 Investigational treatment

<u>Treatment with frequent nocturnal hemodialysis</u>
Patients in this group are included in the study at the start of frequent nocturnal hemodialysis. Frequent nocturnal hemodialysis is defined as hemodialysis sessions of at least 7 hours, performed during 4-7 nights/week. Most often it will be performed as nocturnal home hemodialysis, but patients who perform frequent nocturnal hemodialysis in a dialysis center can also be included. Much experience with this treatment has been obtained, and results are excellent, and complications or technical problems are comparable with conventional hemodialysis[31,32]. Blood flow during hemodialysis is 150 ml/min, dialysate flow is 300 ml/min. Dialysis fluid is different from the fluid in conventional hemodialysis regarding the following constituents: potassium 3 mmol/l, calcium 1.75 mmol/l, bicarbonate 30 mmol/l, phosphate supplementation when necessary.

<u>Treatment with renal transplantation</u>
Patients in this group are included in the study at the moment of renal transplantation, with a living or cadaveric renal transplant. Immunosuppressive medication is given according to the conventional standard regimens, and is adjusted as required by clinical events.

<u>Treatment with conventional hemodialysis</u>
Conventional hemodialysis is defined as hemodialysis sessions of a maximum of 5 hours, mostly 3-4 hours, performed 3-4 times a week. Hemodialysis is applied according to guidelines of the Dutch Nephrology Federation (NFN).

<u>Treatment with peritoneal dialysis</u>
Peritoneal dialysis is defined as treatment with continuous ambulatory peritoneal dialysis (CAPD) or continuous cyclic peritoneal dialysis (CCPD). Peritoneal dialysis treatment is applied according to guidelines of the NFN.

5.2 Co-intervention

<u>Supportive treatment in nocturnal hemodialysis, conventional hemodialysis and peritoneal dialysis</u>
Medications and diet in dialysis patients are prescribed according to the guidelines of the NFN regarding blood pressure regulation, treatment of anemia and acidosis.
Treatment times on hemodialysis will be adjusted when total Kt/V is < 3.6/week. Treatment regimen on peritoneal dialysis will also be adjusted to reach a Kt/V of 1.7/week or 45 L/week.
Regarding calcium and phosphate targets, KDOQI/NFN guidelines are also followed, i.e.:
- target phosphate ≤ 1.78 mmol/l
- calcium phosphate product ≤ 4.4 $mmol^2/l^2$ (with calcium corrected for albumin: correction factor 0.02 mmol/l calcium per 1 g/l decrease in serum albumin below 42 g/l)

- PTH 16-33 pmol/l

In order to improve patient compliance to drug therapy, daily-dose blister packaging will be encouraged in all patients.

Supportive treatment in renal transplant patients

The following medication is given according to common practice:
- daclizumab or basiliximab in case of a second transplant, or in patients with increased immunologic risk
- ranitidine or pantozole during treatment with prednisolone
- cotrimoxazole during the first 4 months post-transplant
- valganciclovir in case of donor CMV IgG + antibodies/ recipient – antibodies
- calcium carbonate as osteoporosis prophylaxis in patients using prednisolone, unless hypercalcemia is present
- amlodipine
- other supportive medication according to common practice when appropriate e.g. for treatment of hypertension, rejection, and other post-transplant complications.

In order to improve patient compliance to drug therapy, daily-dose blister packaging will be encouraged in all patients.

When necessary for medical reasons, the treatment regimen will of course be adjusted to the need of the individual patient.

5.3 Escape medication

Not applicable.

6. INVESTIGATIONAL MEDICINAL PRODUCT

Not applicable.

7. METHODS

7.1 Study endpoints

7.1.1 Main study endpoint

The primary endpoint of the study is:

- change in coronary artery calcification score

7.1.2 Secondary study endpoints

The secondary endpoints of the study are:

- change in pulse wave velocity
- change in coronary artery stenosis
- association between calcium, phosphate, PTH and change in coronary artery calcification score
- association between high-sensitivity-CRP, interleukin-6, interleukin-1, von Willebrand factor, myeloperoxidase, and change in coronary artery calcification score
- association between fetuin A, osteoprotegerin and matrix-Gla protein, and change in coronary artery calcification score
- cardiovascular events and mortality, assessed by the local investigator and the principal investigator separately

7.1.3 Other study parameters

Other parameters that will be studied are:

- body weight
- blood pressure
- laboratory parameters: hemoglobin, cholesterol, HDL-cholesterol, LDL-cholesterol, triglycerides)
- calcification propensity in serum
- quality-of-life questionnaire
- medication (antihypertensive medication, medication concerning calcium-phosphate-homeostasis and immunosuppressive medication)

7.2 Randomization, blinding and treatment allocation

Not applicable.

7.3 Study procedures

The following procedures are no part of medical treatment, thus are extra for this study:

Multislice CT-scanning

Scanning with multi-slice CT is extremely fast. A calcium score scan and a coronary CT scan require less than 10 seconds of time (3 heartbeats). The scans will be performed on a MSCT-scanner with at least 64 detector rows (Brilliance-64, Philips Medical Systems, Best,

The Netherlands). In contrast to contrast-enhanced-CT scans of the heart with a considerable radiation dose, a calcium score multi-slice CT scan allows for a lower image quality and therefore a much lower radiation dose (depending on patient size and gender, radiation exposure will vary between 0.4 and 1.2 mSv, corresponding with less than half a year of natural background radiation). Even when the calcium score scan is combined with a coronary multislice CT-scan, total radiation dose is about 4-5 mSv, which is much less than the radiation dose of a diagnostic CT-scan of the abdomen (10-15 mSv). In some patients a very low dose of beta-blocker (5 mg metoprolol) will be given intravenously when the heart rate is above 60/min, in order to improve imaging of the heart during scanning. This will only be done in patients without contra-indications against beta blockade, which information has to be given to the radiologist by the nephrologist through written confirmation.
To enable assessment of coronary stenosis, the scan will be performed during contrast material injection (40-50 ml of low-osmolar contrast agent), but only in dialysis patients or in patients with adequate renal function (GFR according to MDRD formula ≥45 ml/min).
Protocol calcium score scan: prospectively triggered non-contrast CT, 128 x 0.625 mm collimation, step and shoot technique; reconstruction of 3 mm sections every 1.5 mm; 120 kVp at 50-75 mAs; radiation dose 0.8-1.2 mSv.
Protocol coronary multislice CT: prospectively triggered post-contrast CT, 128 x 0.625 collimation, step and shoot technique; reconstruction of 0.9 mm sections every 0.5 mm; 120 kVp at 150-250 mAs, depending on patient size (weight >60 kg); radiation dose 2.5-4.1 mSv; scanning during injection of 60 ml contrast material (300mg/ml iodine) at flow rate of 6 ml/s, followed by 50 ml normal saline at the same flow rate; short delay will be set to 7 s to ensure optimum contrast enhancement; scan is finished within 3 heart beats.
Risks: nephrotoxicity - but comparatively small dose of contrast material (liver exams, for example, require 150 ml; coronary require 100-300 ml, depending on difficulty of catheterization); contrast allergy - risk for serious reactions: 0.04%; contrast extravasation.

Pulse wave velocity

Pulse wave velocity measurements will be performed at baseline and 1, 2 and 3 years after inclusion. It can be calculated from arterial pulse contours. These are obtained by applanation tonometry (Sphygmo-Cor system, PWV Inc, Sydney, Australia) at the carotid and radial arteries and femoral artery. With this technique the artery is pressed against a hard underground (bone) with a pencil-type probe. The probe contains a strain-gauge transducer at the tip with a small pressure-sensitive ceramic sensor area (Millar Instruments, Houston, Texas, USA). To determine aortic and brachial pulse wave velocity, the distance travelled by the pulse wave over the body surface is measured as the distance between the recording sites at the femoral or radial artery to the suprasternal notch minus the distance from the recording site at the carotid artery to the suprasternal notch, as described by others[33]. The investigation takes about 15-20 minutes.

Quality-of-life questionnaire

Patient's well-being will be assessed at baseline, 1, 2 and 3 years by the Kidney Disease Quality-of-Life Short Form. This questionnaire is validated for dialysis patients[34]. Filling in the questionnaire takes about 15 minutes of time.

The following procedures are part of standard medical treatment:

<u>Venipuncture</u> at baseline and every 12 months, until 3 yr after inclusion. Venipuncture will be combined with venipuncture needed for routine care. Assessment of the routine tests (Hb, lipids, Ca, P, PTH) will be performed in the laboratory of the local treatment center. Assessment of the non-routine tests (inflammatory parameters, calcification inhibitors and serum calcification propensity) will take about 20 cc of blood per 12 month-visit, using sodium citrate and EDTA vacutainers. From February 2017 onwards, additional serum-separating vacutainers will be collected, in order to measure serum calcification propensity in active participants. A protocol concerning collection, processing and storage will be made separately under supervision of the Laboratory for Clinical Chemistry and Hematology Research, University Medical Center Utrecht.

<u>Body weight</u> is assessed at baseline and every 6 months, until 3 yr after inclusion. In transplant patients weight is measured at the ambulant visit. In dialysis patients, dry weight is obtained from the dialysis charts.

<u>Blood pressure</u> is assessed at baseline and every 6 months, until 3 yr after inclusion. In transplant patients and peritoneal dialysis patients, blood pressure is measured at the ambulant visit, in sitting position, by a sphygmomanometer. At least two readings are taken, separated by as much time as is practical; if readings vary by > 5 mmHg, additional readings are taken until two are close[35]. In hemodialysis patients, blood pressure is taken before the hemodialysis session, again at least two readings as described above.

<u>Medication</u> is recorded at baseline and every 12 months, until 3 yr after inclusion: number and defined daily doses of antihypertensive drugs, number and defined daily doses of phosphate binders, number and defined daily doses of vitamin-D-analogues and calcimimetics, number and defined daily doses of immunosuppressive agents.

7.4 Withdrawal of individual subjects

Subjects can leave the study at any time for any reason if they wish to do so without any consequences. The investigator can decide to withdraw a subject from the study for urgent medical reasons.
Patients in the dialysis groups (nocturnal hemodialysis, conventional hemodialysis, peritoneal dialysis) that receive a renal transplant during the study period are asked to undergo a MSCT, pulse wave velocity measurement and laboratory investigation within one month after transplantation. This enables us to assess the primary and secondary endpoint, albeit after a shorter period of time than intended.

7.4.1 Specific criteria for withdrawal

No specific criteria.

7.5 Replacement of individual subjects after withdrawal

After withdrawal, subjects from the transplant group will not be replaced. In the intended number of participants, there is allowed for some withdrawal.

If subjects from the 3 dialysis groups drop out within 18 months from inclusion because of renal transplantation, they are asked to participate in the transplant group, under the following conditions:

* only calcification scores will be measured with MSCT, in order not to exceed the radiation exposure
* if subjects are transplanted within 6 months after inclusion, they will be switched to the transplant group without extra investigations
* if subjects are transplanted after 6 months and before 18 months after inclusion, patients are asked to participate again in the study, in the transplant group.
* a new informed consent must be obtained.

7.6 Follow-up of patients withdrawn from treatment

After withdrawal, follow-up is performed according to the study protocol as far as possible, as depicted in the following diagram, with different examples:

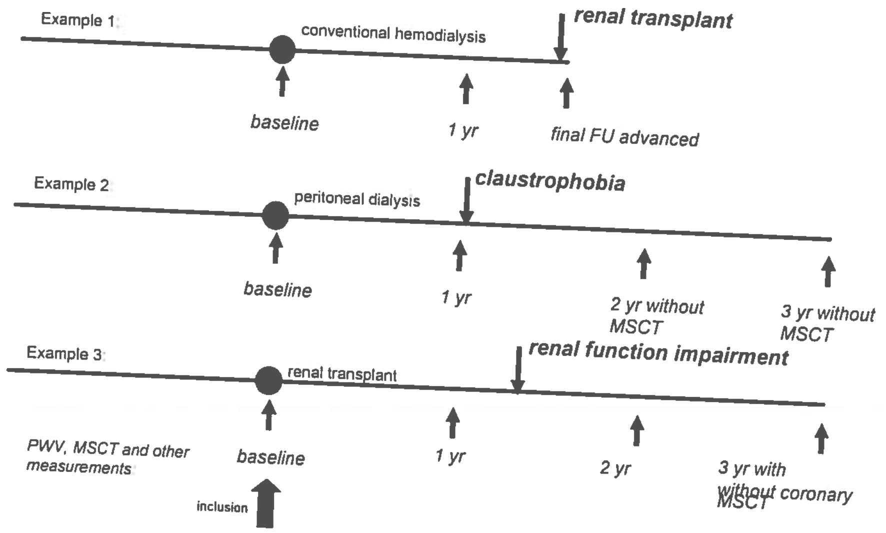

Example 1: when a patient treated with conventional hemodialysis receives a renal transplant after 1 ½ years, for this patient the study will end at 1 ½ years. Now all measurements, including calcium score and coronary MSCT and PWV will be done at 1 ½ years after inclusion, and within one month after the renal transplant.

Example 2: when a patient who is treated with peritoneal dialysis develops claustrophobic symptoms, follow-up is completed according to the study protocol, except for the MSCT.
Example 3: when a patient who is treated with a renal transplant and renal function deteriorates to a GFR less than 30 ml/min, follow-up will be done as planned apart from the coronary MSCT, because contrast medium cannot be given.
Example 4: when a patient treated with conventional hemodialysis receives a renal transplant before 1 ½ years, the patient is asked to participate again in the study; new baseline measurements will be performed, but the coronary MSCT will not be done at inclusion, nor after 3 years. In this case the total radiation exposure will not exceed than the exposure in the original study (maximum of 11.3 mSv).

7.7 Premature termination of the study

The study is an observational study, describing the progression of vessel wall calcification during different accepted treatment modalities. The only reason for terminating the study prematurely is when it would become impossible to measure the primary endpoint, i.e. the coronary artery calcification score. As MSCT is a validated and safe method for measuring calcification, it would be unlikely that this would be necessary. In theory, however, it could be possible in case of severe logistical problems or when new evidence would appear about the safety of MSCT.

8. SAFETY REPORTING

8.1 Section 10 WMO event

In accordance to section 10, subsection 1, of the SMO, the investigator will inform the subjects and the reviewing accredited METC if anything occurs, on the basis of which it appears that the disadvantages of participation may be significantly greater than was foreseen in the research proposal. The study will be suspended pending further review by the accredited METC, except insofar as suspension would jeopardize the subjects' health. The investigator will take care that all subjects are kept informed.

8.2 Adverse and serious adverse events

Adverse events are defined as any undesirable experience occurring to the patient during the study, whether or not considered related to the diagnostic procedures. All adverse events reported spontaneously by the subject or observed by the investigators will be recorded.

A serious adverse event is any untoward medical occurrence or effect that at any dose
- results in death;
- is life threatening (at the time of the event);
- requires hospitalization or prolongation of existing inpatients' hospitalization;
- results in persistent or significant disability or incapacity;
- is a congenital anomaly or birth defect;
- is a new event of the study likely to affect the safety of the subjects, such as an unexpected outcome of an adverse reaction.

As SAEs occur highly frequently in ESRD patients (such as urinary tract infections requiring hospitalization in renal transplant patients), it is infeasible to report every SAE to the METC. SAEs are reported to the accredited METC that approved the protocol according to the requirements of that METC, if a possible relationship to administration of intravenous contrast during coronary MSCT exists, and/or if SAEs occur within 48 hours of a follow-up visit or multislice CT-scan with or without intravenous contrast administration. Deaths are always reported to the accredited METC, irrespective of the conditions mentioned above.

8.2.1 Suspected unexpected serious adverse reactions (SUSAR)

Not applicable

8.2.1 Annual safety report

The annual safety report will be combined with the annual progress report (see chapter 11.3).

8.3 Follow-up of adverse events

All adverse events will be followed until they have abated, or until a stable situation has been reached. Depending on the event, follow up may require additional tests or medical procedures as indicated, and/or referral to the general physician or a medical specialist.

8.4 Data Safety Monitoring Board (DSMB)

No such committee will be established, because the study is non-interventional study, and treatment is not dependent on participation in the study.

9. STATISTICAL ANALYSIS

9.1 Descriptive statistics

The population characteristics are described with a categorical or continuous output, as appropriate.

The primary endpoint, coronary artery calcification score, is a continuous variable with a skewed distribution, and will be presented with its median and ranges. Progression of calcification score will be analyzed with multinomial logistic regression, dividing the coronary artery calcification score in three categories. The same goes for pulse wave velocity of the aorta and iliac arteries.

The calcification score of the different groups will be compared by regression analyses.

9.2 Univariate analysis

The relation between each independent variable (demographic data, calcium-phosphate parameters, inflammation markers and calcification inhibitors), and primary outcomes will be determined with univariate linear regression analysis. The relationship between these independent variables and secondary outcomes (change in coronary calcification score, change in pulse wave velocity) will be determined with univariate linear regression analysis. Because the relative change in calcification score can be independent from the initial absolute calcification score, the results will be corrected for initial calcification score.

9.3 Multivariate analysis

The effects of age, gender, body weight, blood pressure, medication, hemoglobin, lipids, quality-of-life, performance status and medication on the relation between each independent variable (demographic data, calcium-phosphate parameters, inflammation markers and calcification inhibitors), and primary outcomes will be determined with multivariate linear regression analysis. The effects of age, gender, body weight, blood pressure, medication, hemoglobin, lipids, quality-of-life, performance status and medication on the relationship between these independent variables and secondary outcomes (change in coronary calcifications score, change in pulse wave velocity) will be determined with multivariate linear regression analysis.

9.4 Interim analysis

Not applicable

10. ETHICAL CONSIDERATIONS

10.1 Regulation statement

The study will be conducted according to the principles of the Declaration of Helsinki (version 9 October 2004) and in accordance with the Medical Research Involving Human Subjects Act (WMO).

10.2 Recruitment and consent

Patients will be shortly informed about the outline of the study by their physician-in-charge, and asked if they are willing to receive further information. Subsequently, the local investigator or research nurse will provide detailed verbal and written information about the study. The patients will be given at least two weeks to consider participation. Participation is only possible after giving written informed consent, which will be asked by the local investigator.

10.3 Objection by minors or incapacitated subjects

Not applicable

10.4 Benefits and risks assessment, group relatedness

The potential value of this study lies in gaining insight in the process of artery calcification in patients with ESRD. With conventional dialysis methods, progression of arterial calcification occurs alarmingly fast. On theoretical grounds, it is plausible that progression is attenuated in patients with a renal transplant (low/normal phosphate, normal/high calcium) and patients on nocturnal hemodialysis (normal phosphate, normal calcium). However, no long-term data are available on this subject. Therefore it is difficult to advise dialysis patients about registration at the Eurotransplant Kidney Waiting List, especially for patients with advanced age, who have substantial perioperative morbidity and mortality.

The risk and burden to the patient are formed in this study by:

- calcium score MSCT: as described above (7.3 Study procedures) multislice CT is a very short procedure giving a low radiation dose (0.4-1.2 mSv);
- coronary MSCT: as described above (7.3 Study procedures) a coronary CT is also a very short procedure with a low radiation dose (3-4 mSv) (NB for comparison: a diagnostic abdominal CT scan delivers a dose of about 7-10 mSv);
- pulse-wave velocity: non-invasive measurement without risk;
- minor extra blood loss combined with routine venipuncture.

10.5 Compensation for injury

The investigators have a liability insurance which is in accordance with article 7, subsection 6 of the WMO.

An insurance for the patients will be taken out in accordance with the legal requirements in the Netherlands (Article 7 WMO and the Measure regarding Compulsory Insurance for

Clinical Research in Humans of 23th June 2003). This insurance provides cover for damage to research subjects through injury or death caused by the study.
This insurance applies to the damage that will be the result of the study and become apparent during the study or within 4 years after the end of the study.

10.6 Incentives

Subjects who need to travel from home to hospital by own means of transport only for investigations needed for this study, will be compensated by a travel reimbursement (€ 0.25/km plus parking costs, a lunch reimbursement of € 10,-).

11 ADMINISTRATIVE ASPECTS, MONITORING AND PUBLICATION

11.1 Handling and storage of data and documents

Medical information about the patients and data collected for the study are confidential, and will be coded to an individual patient code. The data will only be available for the investigators and medical staff involved in the medical care of the patients.

11.2 Monitoring and quality assurance

The conduct of the study will be monitored. For details we refer to our monitoring plan (see attachment).

11.3 Amendments

Amendments are changes made to the research after a favorable opinion by the accredited METC has been given. All amendments will be notified to the METC that gave a favorable opinion.

11.4 Annual progress report

The investigator will submit a summary of the progress of the trial to the accredited METC once a year. Information will be provided on the date of inclusion of the first subject, numbers of subjects included and numbers of subjects that have completed the trial, serious adverse events/ serious adverse reactions, other problems, and amendments.

11.5 End of study report

The investigator will notify the accredited METC of the end of the study within a period of 8 weeks. The end of the study is defined as the last patient's last visit.
In case the study is ended prematurely, the investigator will notify the accredited METC, including the reasons for the premature termination. Within one year after the end of the study, the investigator will submit a final study report with the results of the study, including any publications/abstracts of the study, to the accredited METC.

11.6 Public disclosure and publication policy

The results of the study will be published in peer reviewed, international medical (nephrology) journals. Possible sponsors will not have influence on the publication of the results, irrespective of their nature.

12. REFERENCES

1. Parfrey PS, Foley RN. The clinical epidemiology of cardiac disease in chronic renal failure. J Am Soc Nephrol 1999;10:1606-1615.

2. Kalantar-Zadeh K, Block G, Humphreys MH, Kopple JD. Reverse epidemiology of cardiovascular risk factors in maintenance dialysis patients. Kidney Int 2003;63:793-808.

3. London GM, Guérin AP, Marchais SJ, et al. Arterial media calcification in end-stage renal disease: impact on all-cause and cardiovascular mortality. Nephrol Dial Transplant 2003;8:731-40.

4. Liu Y, Coresh J, Eustace JA, et al. Association between cholesterol level and mortality in dialysis patients. JAMA 2004;291:451-459.

5. Kalantar-Zadeh K, Block G, Humphresys MH, McAllister CJ, Kopple JD. A low, rather than a high, total plasma homocysteine is an indicator of poor outcome in hemodialysis patients. J Am Soc Nephrol 2004;15:442-453.

6. Block GA, Hulbert-Shearon TE, Levin NW, Port FK. Association of serum phosphorus and calcium x phosphate product with mortality risk in chronic hemodialysis patients: a national study. Am J Kidney Dis 1998;31:607-617.

7. Block GA, Raggi P, Bellasi A, et al.. Mortality effect of coronary calcification and phosphate binder choice in incident hemodialysis patients. Kidney Int. 2007;71:438-41.

8. Ketteler M, Bongartz P, Westenfeld R, et al. Association of low fetuin-A (AHSG) concentrations in serum with cardiovascular mortality in patients on dialysis: a cross-sectional study. Lancet. 2003;361:827-33.

9. Asmus H-G, Braun J, Krause R, et al. Two year comparison of sevelamer and calcium carbonate effects on cardiovascular calcification and bone density. Nephrol Dial Transplant 2005;20:1653-1661.

10. Block GA, Spiegel DM, Ehrlich D, et al. Effects of sevelamer and calcium on coronary artery calcification in patients new to hemodialysis. Kidney Int. 2005;68:1815-24.

11. Goodman WG, Goldin J, Kuizon BD, et al. Coronary-artery calcification in young adults with end-stage renal disease who are undergoing dialysis. N Engl J Med 2000;342:1478-1483.

12. Mucsi I, Hercz G, Uldall R, Ouwendyk M, Francoeur R, Pierratos A. Control of serum phosphate without any phosphate binders in patients treated with nocturnal hemodialysis. Kidney Int 1998;53:1399-404.

13. Pierratos A. Nocturnal home haemodialysis: an update on a 5-year experience. Nephrol Dial Transplant 1999;14:2835-40.

14. Kooistra MP. Frequent prolonged home haemodialysis: three old concepts, one modern solution. Nephrol Dial Transplant 2003; 18:16-9.

15. Yuen D, Pierratos A, Richardson RM, Chan CT. The natural history of coronary calcification progression in a cohort of nocturnal haemodialysis patients. Nephrol Dial Transplant. 2006;21:1407-12.

16. Merx MW, Schäfer C, Westenfeld R, et al. Myocardial stiffness, cardiac remodeling, and diastolic dysfunction in calcification-prone fetuin-A-deficient mice. J Am Soc Nephrol. 2005;16:3357-64.

17. Schafer C, Heiss A, Schwarz A, et al. The serum protein alpha 2-Heremans-Schmid glycoprotein/fetuin-A is a systemically acting inhibitor of ectopic calcification. J Clin Invest. 2003;112:357-66.

18. Luo G, Ducy P, McKee MD, et al. Spontaneous calcification of arteries and cartilage in mice lacking matrix GLA protein. Nature. 1997;386:78-81.

19. Moe SM, Reslerova M, Ketteler M, O'neill K, Duan D, Koczman J, Westenfeld R, Jahnen-Dechent W, Chen NX. Role of calcification inhibitors in the pathogenesis of vascular calcification in chronic kidney disease (CKD). Kidney Int. 2005;67:2295-304.

20. Hermans MM, Brandenburg V, Ketteler M. Association of serum fetuin-A levels with mortality in dialysis patients. Kidney Int. 2007;72:202-7.

21. Hermans MM, Brandenburg V, Ketteler M, Kooman JP, Sande FM, Gladziwa U, Rensma PL, Bartelet K, Konings CJ, Hoeks AP, Floege J. Study on the relationship of serum fetuin-A concentration with aortic stiffness in patients on dialysis. Nephrol Dial Transplant. 2006;21:1293-9.

22. Mehrotra R, Westenfeld R, Christenson P, Budoff M, Ipp E, Takasu J, Gupta A, Norris K, Ketteler M, Adler S. Serum fetuin-A in nondialyzed patients with diabetic nephropathy: relationship with coronary artery calcification. Kidney Int. 2005;67:1070-7.

23. Hermans MM, Vermeer C, Kooman JP, et al. Undercarboxylated Matrix GLA Protein Levels Are Decreased in Dialysis Patients and Related to Parameters of Calcium-Phosphate Metabolism and Aortic Augmentation Index. Blood Purif. 2007 ;25:395-401.

24. Prokop M. Multislice CT: technical principles and future trends. Eur Radiol 2003 Dec;13 Suppl 5:M3-13.

25. Prokop M. The Heart. In: Prokop M, Galanski M (eds). Spiral and multislice computed tomography of the body. Thieme Medical Publishers, Stuttgart, New York 2003: 759-824.

26. Isgum I, van Ginneken B, Olree M. Automatic detection of calcifications in the aorta from CT scans of the abdomen. 3D computer-aided diagnosis. Acad Radiol 2004 Mar;11(3):247-57.

27. Nagel HD, Blobel J, Brix G, Ewen K, Galanski M, Hofs P, Loose R, Prokop M, Schneider K, Stamm G, Stender HS, Suss C, Turkay S, Vogel H, Wucherer M. 5 years of "concerted action dose reduction in CT" -- what has been achieved and what remains to be done? Rofo 2004 Nov;176(11):1683-94.

28. Hager A, Kaemmerer H, Leppert A, Prokop M, Blucher S, Stern H, Hess J. Follow-up of adults with coarctation of the aorta: comparison of helical CT and MRI, and impact on assessing diameter changes. Chest 2004 Oct;126(4):1169-76.

29. Prokop M, Waaijer A, Kreuzer S. CT angiography of the carotid arteries. JBR-BTR 2004 Jan-Feb;87(1):23-9.

30. Haydar AA, Hujairi NM, Covic AA, Pereira D, Rubens M, Goldsmith DJ. Coronary artery calcification is related to coronary atherosclerosis in chronic renal disease patients: a study comparing EBCT-generated coronary artery calcium scores and coronary angiography. Nephrol Dial Transplant. 2004 Sep;19(9):2307-12.

31. Heidenheim AP, Kooistra MP, Lindsay RM. Quality of life. Contrib Nephrol 2004;145:99-105.

32. Kooistra MP. Frequente nachtelijke thuishemodialyse in Nederland: project Nocturne. Eindverslag van het pilotonderzoek. April 2004.

33. Wilkinson IB, Fuchs SA, Jansen IM, et al. Reproducibility of pulse wave velocity and augmentation index measured by pulse wave analysis. J Hypertens 1998;16: 2079-84.

34. Korevaar JC, Merkus MP, Jansen MA, Dekker FW, Boeschoten EW, Krediet RT; NECOSAD-study group. Validation of the KDQOL-SF: a dialysis-targeted health measure. Qual Life Res. 2002 Aug;11(5):437-47.

35. Kaplan NM, Measurement of blood pressure, In: Clinical Hypertension, Williams & Wilkins, 7th ed. 1998 Baltimore.
