# Supplementary material for: Progression of coronary artery calcification in conventional hemodialysis, nocturnal hemodialysis, and kidney transplantation
Source: PLoS One. 2020 Dec 30;15(12):e0244639. doi: 10.1371/journal.pone.0244639 (PMC7773242; doi:10.1371/journal.pone.0244639)
Supplement: Attachment — Submitted filename: Comments.docx [file pone.0244639.s008.docx]

The study enrolled 114 patients on dialysis and divided them into three groups: conventional hemodialysis, nocturnal hemodialysis, and kidney transplantation. The differences of changes of coronary artery calcification (CAC) with three-year period were evaluated using mixed-effect models for these groups. The author concluded that there is no significant difference of CAC progression in the group of kidney transplantation, and nocturnal hemodialysis compared to that in the group of conventional hemodialysis. The advantages of this study include well study design, transparent process in information collection, and correct statistical analysis. However, a main shortage of the study, insufficient sample size, may result in incorrect inference and misguide future research.

In addition, several comments were provided for improvement of this article.

1. I suggest the authors perform post-hoc power analysis. It can estimate type 2 error which represents the probability of false negative conclusion.
2. What are the indications for patients switching therapeutic model from conventional to nocturnal hemodialysis? If the indications are also related to CAC progression, the authors should probably consider to adjust when modeling.
3. Please compare patient characteristics between enrolled and not enrolled cases, which may possibly reflect the limits of generalization.
4. Line 89, Page 4: The sentence, “Patients left the study if they switched renal replacement therapy…” is ambiguous. Dose it also contain that patients switching therapeutic model from conventional to nocturnal hemodialysis? please modify it.
5. I also concern that the lack consistent patient numbers of peritoneal dialysis (n=31) with those (n=40) on previous published on Am J Nephrol 2018;48:369–377 because both number are from NOCTx study. Please explain it.
6. On Page 5, I am not sure whether the description, “dialysis duration defined from the first day to inclusion, minus the time with a functioning kidney transplantation” is correct or not. What does the word “inclusion” mean? Kidney translation should be after the time of inclusion, right?
7. Sum of not enrolled cases in the fig 1 is incorrect, please confirm it.
8. Is there any possible follow-up bias existing in your observation? The lost follow-up rate seems to be higher at 3^rd^ year in the group of conventional hemodialysis than those in the other groups.
9. Does the discrepancies of cardiovascular disease / mortality risk between patients treated with conventional, nocturnal hemodialysis, or kidney transplant recipients probably contributed by the different CAC which reflects potential patient selection at baseline in this study? The author should discuss it.
