# Supplementary material for: Progression of coronary artery calcification in conventional hemodialysis, nocturnal hemodialysis, and kidney transplantation
Source: PLoS One. 2020 Dec 30;15(12):e0244639. doi: 10.1371/journal.pone.0244639 (PMC7773242; doi:10.1371/journal.pone.0244639)
Supplement: Attachment — Submitted filename: Response to reviewers.docx [file pone.0244639.s011.docx]

Thank you for the opportunity to submit a revised version of our manuscript. Please find our responses to the comments below.

**Reviewer 3.**

*1. I must respectfully disagree with reviewer 2 who talks of post hoc power. Power is an estimate used in planning. The data are what they are, and the actual power is either zero or 1 because the effect is either significant or not. I can wholeheartedly recommend the paper "The Abuse of Power" by Hoenig et al in The American Statistician.

The issue of whether there is absence of evidence or evidence of absence is based upon the confidence intervals. The issue in interpretation is whether the extremes of the CI represent something worth knowing about. If, for example, 0.96 is worth knowing about then one cannot rule out a clinically meaningful benefit and the conclusion must be that the study is inconclusive.*

Thank you for pointing this out. We have replaced the paragraph about post-hoc power in the Discussion with a paragraph in which we address that the confidence intervals include a possible clinically meaningful benefit:

*“The 95% confidence intervals of the effect estimates for kidney transplantation include a more than two thirds lower CAC progression rate (adjusted minimum of the 95% confidence interval -0.96), which we consider clinically meaningful.”*

*2. The paper as it stands doesn't adequately explain how people entered one or other group for comparison. What were the selection factors? Clearly there must have been some choice made here and groups cannot be entirely comparable. Nor can those differences be measured exclusively in the variables collected here. Table 1 needs tests for confounding here, and the reason for dialysis vs transplantation in particular is needed.*

The reviewer rightfully addresses the issue of how participants ended up in one treatment group or another, and whether there may be confounder bias beyond the variables presented in Table 1.

In this study, there was no choice made for dialysis versus transplantation. As stated in the Methods and Discussion sections, all patients on dialysis were eligible for transplantation. Hence, the patients on dialysis had not received a kidney transplant, simply because they did not have the luck to receive one yet. Notably, 18 patients on dialysis did have the luck to receive a kidney transplant during study participation.

As for conventional hemodialysis versus nocturnal hemodialysis, there are various reasons why patients opted for nocturnal hemodialysis versus conventional hemodialysis. Important advantages of nocturnal hemodialysis include more free time during the day and less dietary restrictions. As a result, patients opting for nocturnal hemodialysis are often younger, healthier, more motivated, and more likely to adhere to treatment. Although we did not know the reasons of individual patients to opt for nocturnal hemodialysis (which we now acknowledge in the Limitations paragraph of the Discussion), we did adjust for variables that may reflect overall health and thus could influence choice of renal replacement therapy as well as CAC progression (e.g. age, diabetes, and vitamin K antagonist use).

*“For example, we did not know the reasons of individual patients to opt for nocturnal hemodialysis.”*

Following the reviewer’s recommendation, we added significance tests to Table 1. Of note, there were no significant differences in demographics, medical history, and vitamin K antagonist use between treatment groups. There were significant differences in medication use and physical/laboratory parameters, expectedly due to the treatments received. However, we agree with the reviewer that there may be confounding beyond the variables measured in this study. Therefore, we added the following line to the Limitations paragraph of the Discussion:

*“Although there may have been unmeasured confounders, patient groups on dialysis versus kidney transplant recipients were comparable in terms of the measured variables as a result.”*

*3. Please explain the differences in missing data rates in Table 2 - there is potential for bias if these are outcome related.*

The reviewer is right to point out that there were differences in loss to follow-up in our study. Specifically, only 32 out of 57 enrolled patients on conventional hemodialysis completed the first follow-up visit. This could have led to survivor bias. However, only 2 of these 25 patients had died, whereas 4 had withdrawn consent, 7 were lost to follow-up, 7 had received a kidney transplant, and 5 had switched to nocturnal hemodialysis, leaving the study as a result. Therefore, we believe that the high rate of loss to follow-up in this group did not necessarily lead to a selection of healthier patients. We address this issue in the Discussion section, page 18:

*“There was significant loss to follow-up, especially among patients on conventional hemodialysis (25 out of 57 enrolled patients). This could have led to survivor bias. Nevertheless, many patients were lost to follow-up due to kidney transplantation (7/25) or switch to nocturnal hemodialysis (5/25). Therefore, we do not believe that the high rate of loss to follow-up in this group led to a selection of healthier patients.”*
