# Supplementary material for: Progression of coronary artery calcification in conventional hemodialysis, nocturnal hemodialysis, and kidney transplantation
Source: PLoS One. 2020 Dec 30;15(12):e0244639. doi: 10.1371/journal.pone.0244639 (PMC7773242; doi:10.1371/journal.pone.0244639)
Supplement: Attachment — Submitted filename: Response to reviewers.docx [file pone.0244639.s012.docx]

**Response to reviewers**

Thank you for the opportunity to revise our manuscript. Please find our responses to the queries raised by Reviewer #3 below.

--------------------------------------

**Reviewer #3:**

--------------------------------------

*Thank you for your clarification of the design of the study. As I understand it patients will naturally change groups - what is unclear is why patients are excluded for compliance, as this leads to differently selected patients in the different groups.*

We appreciate your concern as to why patients were excluded from the study if they were non-compliant, i.e. non-adherent to dialysis regimes. This was a deliberate choice when we designed the study, for the following. Often, patients initiating nocturnal hemodialysis are younger, healthier, more motivated, and more likely to adhere to treatment compared to the general dialysis population. We therefore sought to minimize these differences by selecting patients on conventional hemodialysis that were somewhat similar in this respect to patients on nocturnal hemodialysis, using treatment adherence as inclusion criterion. Without this criterion, patients on conventional hemodialysis could overall have been less compliant, which theoretically could have led to poorer phosphate control and hence more CAC progression in that group. We therefore believe that rather than introducing bias, this exclusion criterion contributed to reducing bias in our study.

To clarify this, we added the following explanation to the Discussion:

*“On the other hand, we used treatment adherence as an inclusion criterion in order to enroll patients on conventional hemodialysis that were somewhat similar in this respect to patients on nocturnal hemodialysis, who are generally more likely to adhere to treatment”*

--------------------------------------

*[What is unclear is] why patients leave the study on switching therapy as opposed to changing group. The analysis of this sort of study would appear to be the same as one would use for any other transplantation study. It is unclear why Mantel-Byar or equivalent analyses were not used for time to event outcomes and equivalent methodology for multiperiod crossover trials for the other measures.*

We agree with the reviewer that ideally patients would change group rather than leave the study upon switching therapy during study participation, especially in a study with survival as primary endpoint. However, our study did not investigate survival, but had CAC progression as primary endpoint. As a result, it was not feasible for patients to change groups during study participation for the following reasons. First, a switch in therapy would not coincide with the annual follow-up visits during which we measured CAC. Had we decided to allow patients to continue study participation after switching therapy, we would have been left with CAC progression measurements that had been influenced by multiple therapies, which would be difficult if not impossible to interpret. Second, patients would often switch to a hemodialysis therapy that did not match the hemodialysis categories defined in our study (3x4hrs/week or 4-6x8hrs/week). Many would switch to hemodialysis 3x8hrs/week, 4-6x2-4hrs/week, or anything in between. This also precluded continued participation after switching therapy.

For the above reasons, our study did not allow continued participation after switching therapy, and cannot be analyzed as a multiperiod crossover trial. We note that our study did not investigate time-to-event outcomes.

--------------------------------------

*As it stands the different groups appear to enter the study post diagnosis, or time from starting dialysis - how is this allowed for in the analyses as the patients entering after a long period on dialysis are clearly more compliant and have disease that has not led to death (ie could be considered to be more indolent). As it stands the data aren't really interpretable since we are not looking at similar times.*

Thank you for pointing out the issue of patients who have had a long period of dialysis (i.e. dialysis duration or vintage). Patients with a longer dialysis duration are a selected group: mortality has been shown to be the highest in the first 120 days after hemodialysis initiation[Robinson, 2014]. One could thus regard patients with a longer dialysis duration as the “survivors” of the dialysis population – this goes particularly for patients on nocturnal hemodialysis, who generally have had 1-2 years of dialysis before switching to this therapy. This is why we aimed to recruit patients on conventional hemodialysis and kidney transplant recipients who were somewhat similar to patients on nocturnal hemodialysis, using inclusion criteria such as treatment adherence, transplantation eligibility, and a period of dialysis for kidney transplant recipients. Notably, if we look at Table 1, we can appreciate that there were no significant differences in demographics or medical history between the groups, and that dialysis duration was actually quite similar in these three groups (median 27 months in conventional hemodialysis, 29 months in nocturnal hemodialysis, and 28 months in kidney transplant recipients). Thus, patients entered our study after a long period of dialysis, but this was similar for all three therapies. Moreover, we accounted for dialysis duration in our statistical analyses. We therefore do not believe that differences in dialysis duration would have biased our findings with regards to CAC progression.

We now state that the treatment groups were comparable in terms of dialysis duration in the Discussion:

“[..]*We used treatment adherence as an inclusion criterion [and] only enrolled patients on dialysis who were transplantation-eligible [or] kidney transplant recipients that had been on dialysis before transplantation. Although there may have been unmeasured confounders, patient on dialysis versus kidney transplant recipients were [..] comparable in terms of baseline variables, notably age and dialysis duration*”

--------------------------------------

**References**:

Robinson BM, Zhang J, Morgenstern H, et al. Worldwide, mortality risk is high soon after initiation of hemodialysis. Kidney Int. 2014;85(1):158-165. doi:10.1038/ki.2013.252
