# Supplementary material for: Progression of coronary artery calcification in conventional hemodialysis, nocturnal hemodialysis, and kidney transplantation
Source: PLoS One. 2020 Dec 30;15(12):e0244639. doi: 10.1371/journal.pone.0244639 (PMC7773242; doi:10.1371/journal.pone.0244639)
Supplement: Attachment — Submitted filename: Reviewer comments.docx [file pone.0244639.s009.docx]

Thank you for the opportunity to submit a revised version of our manuscript. Please find our responses to the comments below.

**Editor**:

*It is important to include baseline serum calcium, serum phosphorus, plasma PTH levels as covariables in multivariate linear regression.*

We agree with the Editor and Reviewer #2 that it could be valuable to examine the associations of serum and plasma markers of mineral metabolism with CAC progression. However, we should not adjust for these variables when examining the association of renal replacement therapy with CAC progression. The reason is that in the case of renal replacement therapy, these variables are not confounders but mediators; i.e., kidney transplantation and nocturnal hemodialysis could have an effect on CAC progression by changing serum calcium, phosphorus, or plasma PTH[1]. Adjusting for these variables in multivariate regression models would lead to biased estimates instead of removing bias by confounding[2].

*A characteristics comparison between enrolled and non-enrolled cases could be considered to reflect the generalizability.*

We agree with the Editor and Reviewer #2 that it would be valuable to compare characteristics of patients that were enrolled in the NOCTx study (*n=181*) with those who were not (*n=148*). However, as the patients that were not enrolled did not give informed consent for study participation, we could not collect any data on their characteristics. On the other hand, we do compare patient characteristics between enrolled patients that completed follow-up (*n=114*) and those that did not (*n=36*) in the Results section, page 11, lines 184-189:

*“Patients that were excluded from the current analyses as they did not complete any follow-up exam (n=36) had similar characteristics compared to the study population: they were on average 52 ±13 years old, 23 (64%) were male, dialysis duration was median 36 (IQR 18–66) months, and 10 (28%) had diabetes mellitus (all P>0.05 versus study population). Their median CAC score at inclusion was 323 (IQR 1–1181) (P=0.18 versus study population).”*

Also, we have now added a S1 Table comparing further characteristics between enrolled patients that completed follow-up and those that did not.

*The lost follow-up is an important issue in this study that the author need to address this issue.*

The Editor is right to point out that there was significant loss to follow-up in our study. Specifically, only 32 out of 57 enrolled patients on conventional hemodialysis completed the first follow-up visit. This could have led to survivor bias. However, only 2 of these 25 patients had died, whereas 4 had withdrawn consent, 7 were lost to follow-up, 7 had received a kidney transplant, and 5 had switched to nocturnal hemodialysis, leaving the study as a result. Therefore, we believe that the high rate of loss to follow-up in this group did not necessarily lead to a selection of healthier patients. We now address this issue in the Discussion section, page 18:

*“There was significant loss to follow-up, especially among patients on conventional hemodialysis (25 out of 57 enrolled patients). This could have led to survivor bias, although many patients were also lost to follow-up due to kidney transplantation (7/25) or switched to nocturnal hemodialysis (5/25).”*

===================================================

**Reviewer #1:**

*This article entitled “Progression of coronary artery calcification in conventional hemodialysis, nocturnal hemodialysis, and kidney transplantation” by Jansz TT et al investigated progression of CAC between patients treated with different dialysis modality. They concluded that nocturnal hemodialysis and kidney transplantation are not associated with significantly less CAC progression compared to conventional hemodialysis during up to 3 years follow-up. I have several comments as follows.*

*1. This study takes much time and efforts to answer whether nocturnal HD or kidney transplantation decreases vascular calcifications. However, I think that its clinical significance and merit are limited because vascular calcification is not a good surrogate marker (Ref 1 and 2). A good surrogate marker should accurately reflect the clinical event they are supposed to surrogate. However, meta-analysis and randomized controlled studies did not show vascular calcifications reflecting the underlying risk for death in HD patients.*

*3. Because vascular calcification is just a surrogate marker for cardiovascular diseases or related mortality, the authors should explain why this study aims to investigate vascular calcification rather than major adverse cardiovascular events (MACE) or CV-related mortality as the primary outcome.*

Thank you for pointing out this major controversy in the field of cardiovascular disease in end-stage renal disease. We fully agree with the reviewer that it is debatable whether vascular calcification is an adequate surrogate marker for cardiovascular disease. Even more, we believe our data support that vascular calcification may not be an adequate surrogate outcome. As we point out in the Discussion section, there is a discrepancy between our data not showing less vascular calcification progression among kidney transplant recipients, and the fact that kidney transplant recipients have significantly less cardiovascular morbidity and mortality.

Unfortunately, our study was designed in 2008 before these discrepancies came to light. Therefore, our study was designed with CAC progression as primary outcome and was as such not powered for major adverse cardiovascular events or cardiovascular mortality as primary outcome. Nevertheless, we believe that future studies should use major adverse cardiovascular events or cardiovascular mortality instead of vascular calcification as an primary outcome, which we also state in the Discussion section, page 18:

*“Previously, others have also pointed out important discrepancies between the effects of several drugs on vascular calcification and mortality[23]. For example, several trials have demonstrated that drugs such as cinacalcet or sevelamer may slow down progression of coronary artery calcification[24, 25] but lack benefit on mortality[25, 26]. These discrepancies suggest that vascular calcification might only be a secondary phenomenon to vascular damage and may not be harmful in itself. We therefore believe future studies should use* *major adverse cardiovascular events or cardiovascular mortality instead of vascular calcification as primary outcome.”*

*2. The authors mentioned “kidney transplantation is associated with less CAC progression compared to PD” in Discussion. However, I did not find any data in the article supporting this conclusion. Please shows the data or cite the references if available, otherwise please revise this sentence.*

Please accept our apologies for this error. This sentence should have been deleted after a recent redrafting of this manuscript. We have now deleted this sentence accordingly.

*4. The article is not well organized, especially in Introduction and Discussion. I suggest rewrite some paragraphs to highlight the importance of your study. In addition, the repetition of several sentences is found in Discussion. Please revise them.*

As recommended by the reviewer, we revised several paragraphs in the Introduction and Discussion section.

*5. The authors should include baseline serum calcium, serum phosphorus, plasma PTH levels in as covariables in multivariate linear regression. These covariables for vascular calcification are more important compared to residual renal function or Vitamin K antagonists.*

We agree with the Editor and Reviewer #2 that it could be valuable to examine the associations of serum and plasma markers of mineral metabolism with CAC progression. However, we should not adjust for these variables when examining the association of renal replacement therapy with CAC progression. The reason is that in the case of renal replacement therapy, these variables are not confounders but mediators; i.e., kidney transplantation and nocturnal hemodialysis could have an effect on CAC progression by changing serum calcium, phosphorus, or plasma PTH[1]. Adjusting for these variables in multivariate regression models would lead to biased estimates instead of removing bias by confounding[2].

===================================================

Reviewer #2: The study enrolled 114 patients on dialysis and divided them into three groups: conventional hemodialysis, nocturnal hemodialysis, and kidney transplantation. The differences of changes of coronary artery calcification (CAC) with three-year period were evaluated using mixed-effect models for these groups. The author concluded that there is no significant difference of CAC progression in the group of kidney transplantation, and nocturnal hemodialysis compared to that in the group of conventional hemodialysis. The advantages of this study include well study design, transparent process in information collection, and correct statistical analysis. However, a main shortage of the study, insufficient sample size, may result in incorrect inference and misguide future research.
In addition, several comments were provided for improvement of this article.
*1. I suggest the authors perform post-hoc power analysis. It can estimate type 2 error which represents the probability of false negative conclusion.*

As suggested by the reviewer, we performed a post-hoc power analysis. We calculated the sample size needed to detect an effect of the magnitude suggested for kidney transplantation compared to conventional hemodialysis after a 1-year interval by our data, with 80% power and a significance level of 0.05. This yielded a sample size of 359 per group. We now address this issue in the Discussion section, page 18:

*“We performed a post-hoc power analysis, revealing that over 350 patients per group would be needed to detect the difference currently observed between kidney transplant recipients and patients on conventional hemodialysis in a one-year interval with 80% power at a 0.05 significance level.”*

*2. What are the indications for patients switching therapeutic model from conventional to nocturnal hemodialysis? If the indications are also related to CAC progression, the authors should probably consider to adjust when modeling.*

There are various reasons why patients wanted to switch to nocturnal hemodialysis from conventional hemodialysis. Important advantages of nocturnal hemodialysis include more free time during the day and less dietary restrictions. As a result, patients initiating nocturnal hemodialysis in general are often younger, healthier, more motivated, and more likely to adhere to treatment. Although we did not know the reasons of individual patients to switch to nocturnal hemodialysis, we did adjust for variables that may reflect overall health and thus could influence choice of renal replacement therapy as well as CAC progression (e.g. age, diabetes, and vitamin K antagonist use).

*3. Please compare patient characteristics between enrolled and not enrolled cases, which may possibly reflect the limits of generalization.*

We agree with the Editor and Reviewer #2 that it would be valuable to compare characteristics of patients that were enrolled in the NOCTx study (*n=181*) with those who were not (*n=148*). However, as the patients that were not enrolled did not give informed consent for study participation, we could not save any data on their characteristics. On the other hand, we do compare patient characteristics between enrolled patients that completed follow-up (*n=114*) and those that did not (*n=36*) in the Results section, page 11, lines 184-189:

*“Patients that were excluded from the current analyses as they did not complete any follow-up exam (n=36) had similar characteristics compared to the study population: they were on average 52 ±13 years old, 23 (64%) were male, dialysis duration was median 36 (IQR 18–66) months, and 10 (28%) had diabetes mellitus (all P>0.05 versus study population). Their median CAC score at inclusion was 323 (IQR 1–1181) (P=0.18 versus study population).”*

Also, we have now added a S1 Table comparing further characteristics between enrolled patients that completed follow-up and those that did not.

*4. Line 89, Page 4: The sentence, “Patients left the study if they switched renal replacement therapy…” is ambiguous. Dose it also contain that patients switching therapeutic model from conventional to nocturnal hemodialysis? please modify it.*

We apologize for the confusion arising from our wording. Patients in the nocturnal hemodialysis group switched to nocturnal hemodialysis shortly before enrollment. Indeed, if patients switched renal replacement therapy after enrollment (for example from conventional hemodialysis to nocturnal hemodialysis), they left the study. For clarity, we added “after enrollment” to the sentence:

*“Patients left the study if they switched renal replacement therapy after enrollment”*

*5. I also concern that the lack consistent patient numbers of peritoneal dialysis (n=31) with those (n=40) on previous published on Am J Nephrol 2018;48:369–377 because both number are from NOCTx study. Please explain it.*

There is indeed a discrepancy in these numbers. The explanation is that a total of 4 patients were enrolled while on peritoneal dialysis (*n=2*) and conventional hemodialysis (*n=2*), but received a kidney transplantation >6 months after enrollment. These patients therefore had to leave the study, but requested re-enrollment. They were subsequently re-enrolled as kidney transplant recipients. For this paper, we used the data of those 4 patients after kidney transplantation, whereas in the paper Am J Nephrol 2018;48:369–377 we used data of these patients before kidney transplantation. Hence, there is a discrepancy of 2 patients in the peritoneal dialysis group (21 vs 23) and 2 patients in the conventional hemodialysis group (32 vs 34) between this paper and Am J Nephrol 2018;48:369–377.

*6. On Page 5, I am not sure whether the description, “dialysis duration defined from the first day to inclusion, minus the time with a functioning kidney transplantation” is correct or not. What does the word “inclusion” mean? Kidney translation should be after the time of inclusion, right?*

We apologize for the confusion arising from our wording. Patients that were enrolled as kidney transplant recipients had received a kidney transplant shortly before enrollment. We added this to the Methods section, page 4, lines 88-89:

*“NOCTx included [..] patients on dialysis who received a kidney transplant 2-3 months before enrollment.”*

Furthermore, some patients had had kidney transplants longer before but were again on dialysis at time of enrollment. Their time with a kidney transplant was not counted towards dialysis duration. When mentioning inclusion in this context, we meant enrollment. For clarity, we replaced the word inclusion with enrollment throughout the manuscript.

*7. Sum of not enrolled cases in the fig 1 is incorrect, please confirm it.*

Please accept our apologizes for this error. The number of patients with “other reasons” should be 57 instead of 56. We have amended the figure accordingly.

*8. Is there any possible follow-up bias existing in your observation? The lost follow-up rate seems to be higher at 3rd year in the group of conventional hemodialysis than those in the other groups.*

Thank you for addressing this issue. Indeed, the rate of loss to follow-up was higher in the conventional hemodialysis group than in the nocturnal hemodialysis or kidney transplantation group. Notably, more than a third of the patients on conventional hemodialysis (34%) were lost to follow-up because they received a kidney transplant, which we mention in the Results section, page 11. We now also acknowledge this issue in the Discussion section, page 18:

*“There was significant loss to follow-up, especially among patients on conventional hemodialysis”*

*9. Does the discrepancies of cardiovascular disease / mortality risk between patients treated with conventional, nocturnal hemodialysis, or kidney transplant recipients probably contributed by the different CAC which reflects potential patient selection at baseline in this study? The author should discuss it.*

We thank the reviewer for addressing the issue of potential selection bias. Our study is observational and as such cannot fully eliminate potential selection bias. We attempted to minimize this by only enrolling patients on dialysis that were eligible for kidney transplantation and kidney transplant recipients that had been on dialysis. As a result, the patient groups were similar e.g. in terms of age and dialysis duration, although diabetes mellitus was somewhat more frequent in patients on conventional hemodialysis versus kidney transplant recipients (6/32 versus 3/48, P=0.22), which we adjusted for in our statistical analyses. We now discuss this issue in the limitations paragraph of the Discussion:

*“Finally, our study was observational, which could potentially lead to selection bias. To minimize this, we only enrolled patients on dialysis who were transplantation-eligible and kidney transplant recipients that had been on dialysis before transplantation. The patient groups were comparable as a result”*

**References:**

1. Rocco MV, Lockridge RS Jr, Beck GJ, Eggers PW, Gassman JJ, Greene T, Larive B, Chan CT, Chertow GM, Copland M, Hoy CD, Lindsay RM, Levin NW, Ornt DB, Pierratos A, Pipkin MF, Rajagopalan S, Stokes JB, Unruh ML, Star RA, Kliger AS; Frequent Hemodialysis Network (FHN) Trial Group, Kliger A, Eggers P, Briggs J, Hostetter T, Narva A, Star R, Augustine B, Mohr P, Beck G, Fu Z, Gassman J, Greene T, Daugirdas J, Hunsicker L, Larive B, Li M, Mackrell J, Wiggins K, Sherer S, Weiss B, Rajagopalan S, Sanz J, Dellagrottaglie S, Kariisa M, Tran T, West J, Unruh M, Keene R, Schlarb J, Chan C, McGrath-Chong M, Frome R, Higgins H, Ke S, Mandaci O, Owens C, Snell C, Eknoyan G, Appel L, Cheung A, Derse A, Kramer C, Geller N, Grimm R, Henderson L, Prichard S, Roecker E, Rocco M, Miller B, Riley J, Schuessler R, Lockridge R, Pipkin M, Peterson C, Hoy C, Fensterer A, Steigerwald D, Stokes J, Somers D, Hilkin A, Lilli K, Wallace W, Franzwa B, Waterman E, Chan C, McGrath-Chong M, Copland M, Levin A, Sioson L, Cabezon E, Kwan S, Roger D, Lindsay R, Suri R, Champagne J, Bullas R, Garg A, Mazzorato A, Spanner E, Rocco M, Burkart J, Moossavi S, Mauck V, Kaufman T, Pierratos A, Chan W, Regozo K, Kwok S. The effects of frequent nocturnal home hemodialysis: the Frequent Hemodialysis Network Nocturnal Trial. Kidney Int. 2011 Nov;80(10):1080-91.
2. Jager KJ, Zoccali C, MacLeod A, Dekker FW. Confounding: what is it and how to deal with it. Kidney Int 2008 Feb;73(3):256-60.
